# Plant height measurement using UAV-based aerial RGB and LiDAR images in soybean

**DOI:** 10.3389/fpls.2025.1488760

**Published:** 2025-01-30

**Authors:** Lalit Pun Magar, Jeremy Sandifer, Deepak Khatri, Sudip Poudel, Suraj KC, Buddhi Gyawali, Maheteme Gebremedhin, Anuj Chiluwal

**Affiliations:** College of Agriculture, Health, and Natural Resources, Kentucky State University, Frankfort, KY, United States

**Keywords:** soybean, plant height, high throughput aerial phenotyping, unmanned aerial vehicles, RGB, lidar

## Abstract

Phenotypic traits like plant height are crucial in assessing plant growth and physiological performance. Manual plant height measurement is labor and time-intensive, low throughput, and error-prone. Hence, aerial phenotyping using aerial imagery-based sensors combined with image processing technique is quickly emerging as a more effective alternative to estimate plant height and other morphophysiological parameters. Studies have demonstrated the effectiveness of both RGB and LiDAR images in estimating plant height in several crops. However, there is limited information on their comparison, especially in soybean (*Glycine max* [L.] Merr.). As a result, there is not enough information to decide on the appropriate sensor for plant height estimation in soybean. Hence, the study was conducted to identify the most effective sensor for high throughput aerial phenotyping to estimate plant height in soybean. Aerial images were collected in a field experiment at multiple time points during soybean growing season using an Unmanned Aerial Vehicle (UAV or drone) equipped with RGB and LiDAR sensors. Our method established the relationship between manually measured plant height and the height obtained from aerial platforms. We found that the LiDAR sensor had a better performance (R^2^ = 0.83) than the RGB camera (R^2^ = 0.53) when compared with ground reference height during pod growth and seed filling stages. However, RGB showed more reliability in estimating plant height at physiological maturity when the LiDAR could not capture an accurate plant height measurement. The results from this study contribute to identifying ideal aerial phenotyping sensors to estimate plant height in soybean during different growth stages.

## 1 Introduction

Soybean (*Glycine max* (L.) Merrill) is a vital source of oil and plant protein globally, recognized for its high nutritional value (Wilcox, 2016). In the U.S., it ranks as the second most cultivated crop, following corn (Vaiknoras and Hubbs, 2023), and it plays a significant role in agricultural exports, with the U.S. being the second-largest soybean exporter, accounting for 38% of global soybean trade. To meet the growing global demand and maintain its status as a top exporter, the U.S. must significantly enhance soybean yield. Like other crops, yield in soybean is significantly influenced by a complex interaction of genetic traits, environmental factors, and agricultural practices. Key yield-related traits like pod number and seeds per pod (Ning et al., 2018), seed size (Liu et al., 2011), plant architecture like plant height (Jin et al., 2010), phenology (Kantolic and Slafer, 2001), photosynthetic efficiency (Wang et al., 2023), reproductive efficiency (Tischner et al., 2003) and nitrogen fixation efficiency (Imsande, 1992) are critical to influence final yield. Among these traits, plant height (PH) is one of the main critical yield-related traits in soybean, impacting the crop’s ability to compete for light and, consequently, its overall productivity (Gawęda et al., 2020). Defined as the distance from the ground to the top of the primary photosynthetic tissue (Cornelissen et al., 2003), PH impacts essential factors such as biomass (Bendig et al., 2014; Tilly et al., 2015; Brocks and Bareth, 2018), crop yield (Yin et al., 2011; Sharma et al., 2016; Zhang et al., 2017), and soil nutrient availability (Yin and McClure, 2013). This makes it a pivotal trait in plant breeding and crop improvement programs. Traditionally, measuring PH involves using rulers in the field, a method that is labor-intensive, time-consuming, and susceptible to errors, especially over extensive areas. These manual measurement techniques also suffer from spatial and temporal limitations that can compromise the accuracy of this vital plant phenotype data. To address these challenges, non-destructive image-based phenotyping has become increasingly popular, providing a more efficient and accurate means to assess PH.

Advancements in remote sensing have led to the exploration of various sensor-based methods for effective PH assessment. Passive sensors like satellites have been explored to measure PH in forests (Petrou et al., 2012) and crops like corn (Gao et al., 2013) and rice (Erten et al., 2016). Cloud cover and the revisit time of satellites (Zhang et al., 2020) can limit their effectiveness in precision agriculture. In response to these limitations, recent decades have seen a shift towards proximal field phenotyping technologies. Devices such as ultrasonic sensors, RGB depth cameras, and Terrestrial laser scanners fitted in fixed platforms, tractors, or autonomous robots have become prominent for high throughput field phenotyping. These technologies have proven successful in various crops, including cotton (Jiang et al., 2016; Sun et al., 2017, 2018; Thompson et al., 2019), corn (Hämmerle and Höfle, 2016; Qiu et al., 2019), and soybean (Ma et al., 2019). However, these proximal sensor platforms face several challenges including high cost, limited area coverage, and reduced mobility as the crops reach advanced stages of growth (Deery et al., 2014).

To further enhance the scope and efficiency of phenotyping, high throughput aerial phenotyping (HTAP) using unmanned aerial vehicles (UAV) like drones has gained popularity. UAVs, equipped with various sensors provide rapid, extensive data collection capabilities. Among these, RGB-based photogrammetry has become a popular technique for estimating PH across different crop species, including cotton (Xu et al., 2019; Ye et al., 2023), wheat (Madec et al., 2017; Khan et al., 2018; Yuan et al., 2018; Volpato et al., 2021), maize (Han et al., 2018; Malambo et al., 2018; Su et al., 2019; Gao et al., 2022; Liu et al., 2024) and sorghum (Watanabe et al., 2017; Tunca et al., 2024). UAV-based RGB cameras are popularly used to estimate PH using the structure from motion (SfM) technique (Kalacska et al., 2017; Coops et al., 2021). The PH estimation techniques using RGB cameras are considered a low-cost and user-friendly approach (Li et al., 2019). However, some studies argued that the derived canopy height from the SfM technique showed some issues in height measurement (Cunliffe et al., 2016; Wijesingha et al., 2019). The overestimation of the digital surface model (DSM) by the RGB camera is attributed to its inability to penetrate the canopy and give precise information (Madec et al., 2017). Hence, the LiDAR technique is more popular for vertical structure measurement as its pulses have powerful penetration capacity (Lefsky et al., 2002). LiDAR is particularly noted for its capacity to provide detailed 3D structural information by penetrating dense canopies and differentiating between ground and non-ground points using multiple reflections of laser pulses (Calders et al., 2020; Coops et al., 2021). This technology has effectively estimated canopy height in forests, shrubs, and various crops (Liu et al., 2018; Zhao et al., 2022). Additionally, this technology has successfully predicted PH in many crops like cotton (Sun et al., 2017, 2018; Thompson et al., 2019), wheat (Madec et al., 2017; Yuan et al., 2018; Guo et al., 2019; ten Harkel et al., 2019; Blanquart et al., 2020), maize (Andújar et al., 2013; Zhou et al., 2020; Gao et al., 2022), sorghum (Hu et al., 2018; Wang et al., 2018; Waliman and Zakhor, 2020; Patel et al., 2023) and rice (Tilly et al., 2014a; Phan and Takahashi, 2021; Sun et al., 2022; Jing et al., 2023).

Despite these advancements, there remains a gap in comprehensive UAV-based HTAP studies specifically for estimating soybean height. Previous studies using an imaging system of RGB camera and photonic mixer detector (PMD) have provided valuable insights into PH in a controlled setting (Guan et al., 2018; Ma et al., 2019). Structure from motion (SFM) techniques yield PH that helps spot ideotype in soybeans (Roth et al., 2022). Canopy height and their temporal changes across the growing season were recorded using RGB imagery captured with a drone in different soybean cultivars (Borra-Serrano et al., 2020). Reliable information about soybean height was found when using a low-cost depth camera mounted on a ground-based phenomics platform (Morrison et al., 2021). This study further verified the PH information using information recorded in the field manually and using single-point LiDAR (SPL) with high precision, assuring the ability of LiDAR to perform precise PH estimation in soybeans. Similarly, Luo et al. (2021) recorded data using UAV-based LiDAR and explored the potential of UAV-based LiDAR sensors to estimate soybean height. To our knowledge, no other studies have used UAV-LiDAR multiple times to measure PH in soybeans. Furthermore, none of the previous studies compared the effectiveness of UAV-based LiDAR and RGB for PH estimation in soybean. As a result, there is not yet clear information regarding which aerial phenotyping sensors and timing are ideal for assessing PH in soybean. Hence, in this study, we used UAV-based RGB cameras and LiDAR sensors in the same field experiment to estimate soybean plant height across different periods. This study aims to evaluate the potential of UAV-based RGB and LiDAR sensors for accurately estimating soybean height at different growth and developmental stages. Hence, the objectives of this study is to assess the uncertainty in estimating soybean height with UAV-based RGB and LiDAR sensors and identify the best high throughput aerial phenotyping sensor for PH estimation in soybean.

## 2 Materials and methods

### 2.1 Experimental design

**Figure 1:**
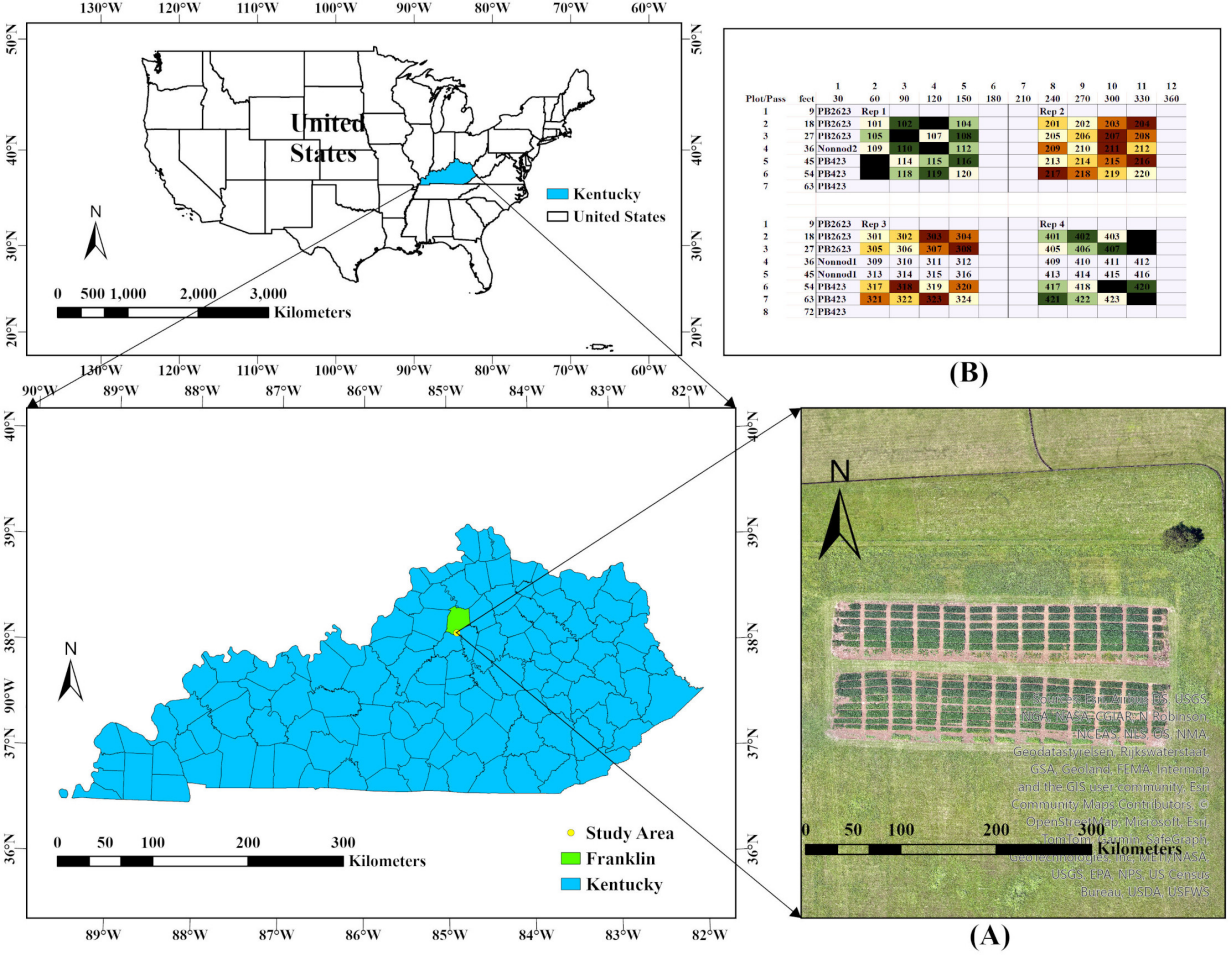
Study area location and experimental design: **(A)** Experimental area location and **(B)** Experimental design in the field.

A field experiment was conducted in 2023 soybean growing season at Kentucky State University’s Harold R. Benson Research and Demonstration Farm (38^0^7^′^ N; -84^0^53^′^ W; and 207 masl). The experiment was set up at Split-Split-Plot Randomized Complete Block Design with four replications (**Figure 1**). The main plot was biochar application: no application or biochar application at 12 tons/ha before planting. Four soybean genotypes (two commercial cultivars - PB2623, PB423, and two advanced breeding non-nodulating soybean lines - KS4120NSGT and KS4120NSGT_NN_NIL-268) were used in the experiment as the subplot. Similarly, sub-subplots were four different levels of late-season N fertilization: 0, 40, 80, and 120 kg N ha^-1^. There were 88 plots, each measuring 7.32 m (24 ft) long and 1.83 m (6 ft) wide, with a 90 cm (3 ft) alley separating the plots. Each plot had five rows and was 38 cm (15 inches) apart. Urea was used as N fertilizer, which was equally split into 3 doses at R5, 1 week after R5, and 2 weeks after R5. Soybean was planted on mid-May and harvested on the last week of September.

### 2.2 Data acquisition

#### 2.2.1 UAV data

The UAV-DJI Mavic 3M (DJI Technology Co., Ltd., Shenzhen, China) fitted with RGB and Multispectral sensor (MS), was employed to capture aerial images of crops. The imaging sensor used was a 1/2.8 -inch CMOS with a 25 mm focal length, capturing images at a resolution of 5280 x 3956 pixels. Drone Deploy was utilized to identify the target area for aerial photography on a satellite map and to plan the flight route by entering the necessary flight and camera parameters. Additionally, the DJI Matrice 300 fitted with Zenmuse L1 LiDAR sensor was utilized to gather aerial LiDAR data. **Table 1** presents the detailed parameters of the drones used in the study. These aerial operations were conducted during solar noon- between 10 AM and 2 PM for optimal lighting conditions, reduced shadows, and uniform illumination. The UAV maintained a consistent altitude of 150 feet above ground level and a flight speed of 5 miles per hour throughout the aerial data collection period.

**Table 1:** Detailed parameter settings of the unmanned aerial vehicles.

Parameter	DJI Mavic 3M	DJI Matrice 300
Relative flight altitude	150 feet	150 feet
Flight speed	5 miles per hour	5 miles per hour
Forward overlap rate	85%	85%
Side overlap rate	85%	85%
Sensor	Multispectral and RGB	L1 LiDAR sensor

Data collection with the UAV-LiDAR and RGB images occurred on June 6, July 7, July 18, and August 29. The first flight generated the reference ground to compute the digital terrain model (DTM). The rest of the dates correspond to critical growth stages of the crop, providing essential data for monitoring its development.

Six ground control points (GCPs) were set up and evenly distributed in the field, with three GCPs on each side. R12 Trimble (Trimble Inc., Sunnyvale, California) recorded the positions of the GCPs and checkpoints.

#### 2.2.2 Field data

**Figure 2:**
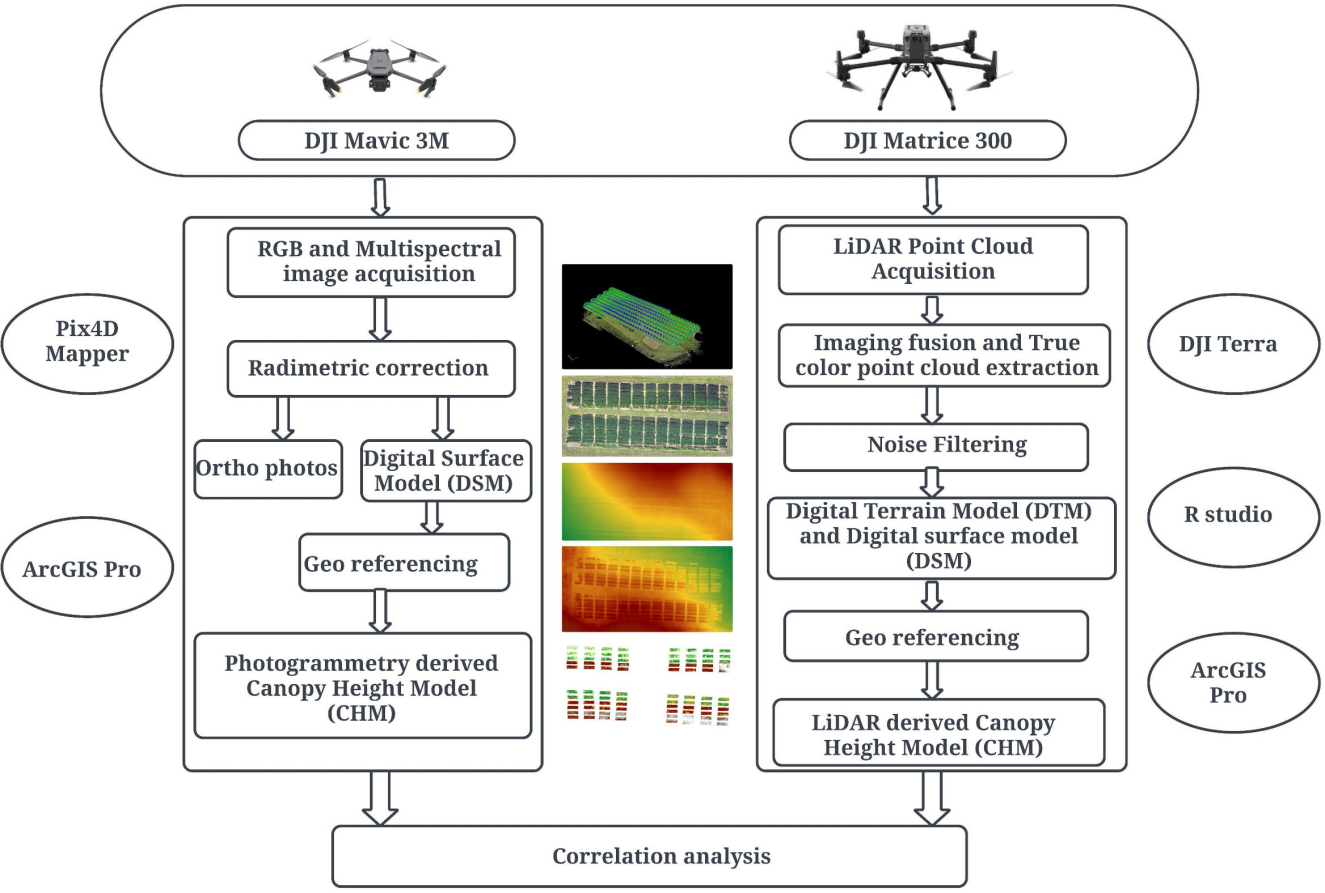
Workflow of the study showing data acquisition, data processing, and data analysis.

In the field, the PH was measured manually from the base to the tip of the main stem to serve as the ground reference height. Within each plot, three sampling locations were randomly chosen, each consisting of one plant from middle three rows to reduce border effect. This sampling approach ensures sufficient representation of plot level crop height (Dhami et al., 2020). The height of these plants was measured using a 1-meter ruler and the results were documented in a field notebook. Height measurements were taken on July 7, July 18, and August 29, aligning with key developmental stages of the soybean: R3 (beginning of pod development), R5 (onset of seed filling), and R7 (start of physiological maturity of the pod). **Figure 2** shows the further steps carried out after the acquisition of the aerial data and field data.

### 2.3 UAV data processing

In the Pix4Dmapper (Pix4D, Lausanne, Switzerland), the captured RGB images were geometrically corrected through orthorectification and stitched together using mosaicking techniques. Orthorectification ensures spatial consistency by correcting geometric distortions caused by terrain variation and camera orientation (Toutin, 2004). Similarly, high overlap rates help to provide repetition in image alignment and reduce gaps and mismatch in processed images (Turner et al., 2012; Colomina and Molina, 2014). These two important steps were instrumental to minimize splicing errors during mosaicking technique. These processes were carried out in Pix4Dmapper recognized for its precision in photogrammetric technique (Gonçalves and Henriques, 2015). Ultimately, the digital surface model (DSM) and orthophotos were then generated using the software’s structure from motion (SFM) techniques.

The raw LiDAR point clouds collected by the UAV were uploaded to DJI Terra software (DJI Technology Co., Ltd., Shenzhen, China), where noise filtering was conducted. The data were formatted into LAS files with specified output coordinates. These LAS files were further post-processed in R studio using the lidR package, which involved setting up scan angle, ground classification, and normalizing elevations to produce the final Digital terrain model (DTM) and DSM.

The generated DTM and DSM were imported into ArcGIS Pro (Esri Inc., Redlands, United States). Here, each image was georeferenced using the georeferencing tool to add control points, utilizing the x and y locations of 6 ground control points (GCPs) and 14 checkpoints. Subsequent elevation adjustments were made to ensure accuracy in the final geographic positioning and elevation data.

### 2.4 Establishment of ground or terrain reference

We used the earliest dates of lidar data we had, classified ground points, filtered for ground class only, and used these to represent the bare earth surface. We then used the ground control points (or GCPs) to establish the adjustment needed to offset the generated DTM elevation to match the actual elevation recorded by the GCPs. We had several GCPs on bare or near-bare earth to measure the deviation between these points and the actual GCPs, thereby having the adjustment needed to match the elevations. All the while ensuring adherence to a projected KY StatePlane coordinate reference system.

The adjustment performed is primarily to correct an error introduced first using different takeoff locations (i.e., different elevations) and the proclivities of the GPS-equipped devices to slightly misjudge the vertical elevation of the takeoff locations (i.e., 234.5 msl versus 245.1 msl recorded at the same spot) on any given day. While the global baselines are impacted, the relative elevation variation in our image is not affected before normalization. The corrected images were further georeferenced, and elevation adjustment was performed to align our images to accurate terrain before generating plot-level data.

In the case of RGB images, the earliest date from the RGB camera was used to generate a bare earth surface using the photogrammetry technique in the Pix4Dmapper. After generating terrain in the software, georeferencing and elevation adjustment were conducted in the ArcGIS Pro software.

### 2.5 Determination of optimum LiDAR scan angle and elevation adjustment

The LiDAR scan angle, which is the angle at which laser pulses are directed toward the ground, plays a crucial role in generating a precise DTM using a LiDAR sensor. The study focused on minimizing the scan angle as closer angles to zero tend to yield more accurate elevation data (Ehlert and Heisig, 2013). To fine-tune our methodology, we conducted several trials to determine the most effective scan angle ranges. We aimed to select angles close to zero that still provided precise elevation information to generate accurate DTM.

**Figure 3:**
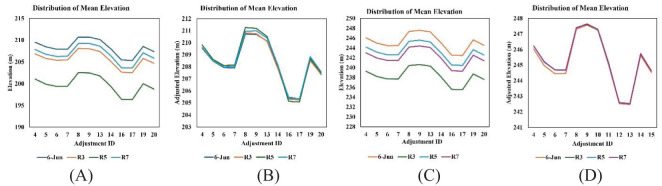
Distribution of mean elevation values across different GCPs and checkpoints: **(A)** before adjustment (RGB images); **(B)** after adjustment (RGB images); **(C)** before adjustment (LiDAR images); **(D)** after adjustment (LiDAR images).

Terrain irregularities and slope variations can significantly affect the accuracy of height measurements from aerial data (Smith et al., 2019). As our study field had irregular terrain, elevation adjustment was carried out. To adjust the elevation values, buffers were created around adjustment points in the field. The mean elevation value of the buffers was computed, and the offset values were generated, which were further used to adjust the DTM and DSM raster. **Figure 3** illustrates the distribution in mean elevation values before adjustment and corrected mean elevation values after adjustment across various GCPs and checkpoints used as adjustment IDs.

This step ensures that all raster accurately represent the terrain by aligning them with actual elevation data. This process focuses on ensuring the elevation data within the raster was precisely adjusted, providing an accurate base for further analysis, like creating a CHM from adjusted DTM and DSM.

### 2.6 DTM, DSM, and CHM extraction

For this study, we initially generated a bare-ground Digital Terrain Model (DTM) by employing photogrammetry techniques to process RGB imagery captured during the early growth phase on June 6. Following this, Digital Surface Models (DSM) were created using RGB imageries acquired from UAV flights during reproductive stages. We integrated and spatially aligned the DTM and DSMs using Ground Control Points (GCPs) within ArcGIS Pro.

For LiDAR point clouds, the DTM was constructed by isolating ground points from the dense point cloud data and interpolating between these points to form a continuous ground elevation model. As outlined by Evans and Hudak (2007), the multiscale curvature classification algorithm facilitated the differentiation of ground and non-ground points. These points were further refined using a Triangulated Irregular Network (TIN) algorithm to produce the DTM for the LiDAR data recorded during the initial crop growth stage. Finally, the ‘pixel metrics’ function from the lidR package was used to compute the DTM value from the LiDAR point cloud.

The DSM for subsequent dates utilized the same function (pixel metrics) to calculate each pixel’s minimum, maximum, and difference. Typically, the maximum value represents the DSM and was utilized to compute CHM after georeferencing and elevation adjustment in ArcGIS Pro. We computed the CHM of the soybean crop in ArcGIS Pro using the raster calculator tool, which involved subtracting the DTM from the DSM at the pixel level. We further refined the CHM at the plot level using the ‘extract by mask’ tool, integrating this with the plot shapefile feature class containing 88 identical plot shapes. Then, plot-level statistics were derived using the ‘zonal statistics as a table’ as a spatial analyst tool. Through this methodical approach, we efficiently generated CHM data from both RGB images and LiDAR point cloud data in ArcGIS Pro.

### 2.7 Statistical analysis

The PH data derived from RGB, LiDAR, and manual measurements were analyzed using simple linear regression. In the simple linear regression model, manually measured PH was considered the dependent variable and sensor-based PH was used as an explanatory variable. We validated the soybean heights estimated from RGB and LiDAR against the manually measured PH. To evaluate the accuracy of these estimations, we calculated the coefficient of determination (R^2^), root mean square error (RMSE), and mean absolute error (MAE), which were computed to see the accuracy of LiDAR and RGB for estimating PH. The R^2^ value assessed how closely the estimated values aligned with the measured values with higher R^2^ values indicating a better fit. Conversely, lower RMSE and MAE values suggested greater accuracy in the estimates, quantifying the difference between the estimated and actual values. The formulas for calculating R^2^, RMSE, and MAE are provided to ensure a clear understanding of how these metrics are derived and interpreted.


$$R^{2}=\frac{\sum_{i=1}^{N}{\left(y_{i}-\hat{y_{i}}\right)}^{2}}{\sum_{i=1}^{N}{\left(y_{i}-{\bar{y}}_{i\;}\right)}^{2}}$$

$$RMSE=\sqrt{\frac{\sum_{i=1}^{N}{\left(y_{i}-\hat{y_{i}}\right)}^{2}}{N}}$$

$$MAE=\frac{1}{N}\sum_{i=1}^{N}|y_{i}-\hat{y_{i}}|$$

Where N is the number of samples, $y_{i}$ and $\hat{y_{i}}$ are the measured and estimated PH, $\left|y_{i}-\hat{y_{i}}\right|$ is the absolute error for the i−th data, respectively ${\bar{y}}_{i\;}$ is the average measured PH.

### 2.8 Modeling calibration and validation

Initially we assessed the individual sensor performance using a simple linear regression method. Later, we employed a variety of regression models, including multiple linear regression (MLR), partial least square regression (PLSR), random forest (RF), and Gaussian process regression (GPR) by integrating both RGB and LiDAR dataset to enhance predictive accuracy by leveraging the strength of both datasets. The dataset was split into training (80%) and testing (20%) sets using random partition method. The splitting was performed at plot level, so the training plots did not include any measurements from test plots. The training set was used to train the models, and the testing set was used to evaluate the performance metrics. The MLR and PLSR models were implemented using linear techniques, while RF and GPR were used for non-linear predictions. Each model was trained on the training dataset using the ‘caret’ package in R Studio. The models’ performance was evaluated using three metrics: R^2^, RMSE, and MAE. 5-fold cross-validation was performed to assess the generalizability of the models. The parameter configuration for each model is clearly explained in **Table 2**.

**Table 2:** Parameters and description of various regression models.

Model	Parameter	Description
Multiple Linear Regression (MLR)	Predictors	RGB, LiDAR
	Coefficient	Automatically estimated using ordinary least squares (OLS)
	Assumptions	Linearity, independence, homoscedasticity, and normality of residuals
Partial Least Square Regression (PLSR)	Number of components	Selected: 3 (via 5 fold cross validation)
Random Forest (RF)	Number of trees (ntree)	Default: 500
	Number of variables (mtry)	Selected: 1 (via 5 fold cross validation)
	Node size	Default: 5
Gaussian Process Regression (GPR)	Kernel type	Radial Basis Function (RBF) kernel (gaussprRadial method in caret)
	Hyperparameters	Optimized via default settings in the caret package

## 3 Results

### 3.1 Estimation of scan angle for the soybean PH study

**Figure 4:**
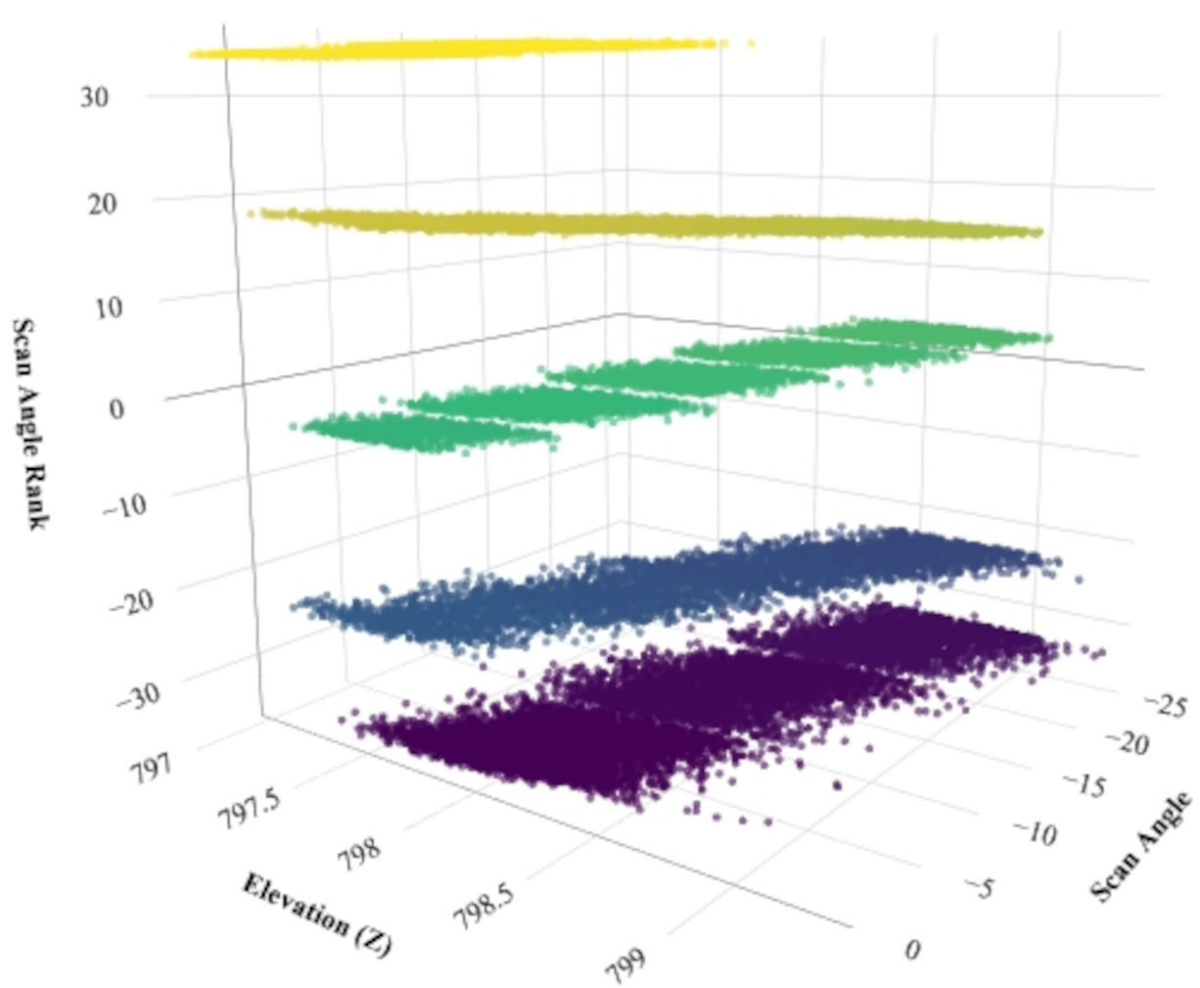
Distribution of LiDAR pulses across different scan angles in various elevations within a transect in the experimental field.

The point closer to the nadir tends to provide a consistent elevation reading as the LiDAR pulses hit the ground more directly (closer to perpendicular), reducing the distortions. In our study, the most consistent and precise capture of the ground elevation appears to occur within the scan angle range of -15 to +15 for DTM generation, as shown in **Figure 4**. The distribution of the LiDAR pulses within this range is more tightly clustered, indicating the consistency in elevation information with reduced variability. When looking at the LiDAR pulses distribution across other angles farther from the nadir, more outlier values appeared in the dataset.

### 3.2 Elevation adjustment

**Figure 5:**
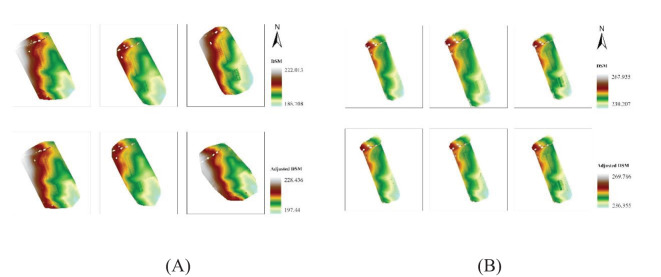
Digital surface model generated from **(A)** RGB and **(B)** LiDAR sensors on different dates. The upper plot layout shows the DSM before elevation adjustment, and the lower plots shows the adjusted DSM.

DSM and DTM raster were adjusted to locate the aerial images to their absolute position in the ground. Using the offset values generated around the GCPs and checkpoints, the raster created using both RGB and LiDAR platforms was adjusted. The shift in the elevation values in RGB and LiDAR-generated DSM was noticed after correcting them using adjustment values. In the case of the DSM generated using an RGB camera, the uppermost elevation values shifted to 228.436 meters from 222.013 meters (**Figure 5A**). In the DSM generated from aerial LiDAR, the uppermost elevation value shifted to 269.786 meters in the adjusted DSM adjusted DSM (**Figure 5B**).

### 3.3 Ground reference height

**Table 3** shows the results from the manual height measurement in the field. It shows the descriptive data of field-measured PH conducted across 88 plots at three different soybean growth and developmental dates. The average PHs recorded on July 7, July 18, and August 29 were 0.39 meters, 0.65 meters, and 0.88 meters, respectively.

**Table 3:** Descriptive statistics of ground reference height.

Date	Mean	Standard deviation	Minimum	Maximum
7-Jul	0.385	0.066	0.23	0.5
18-Jul	0.645	0.11	0.385	0.86
29-Aug	0.874	0.113	0.61	1.12

### 3.4 Estimation of crop height using CHM

**Figure 6:**
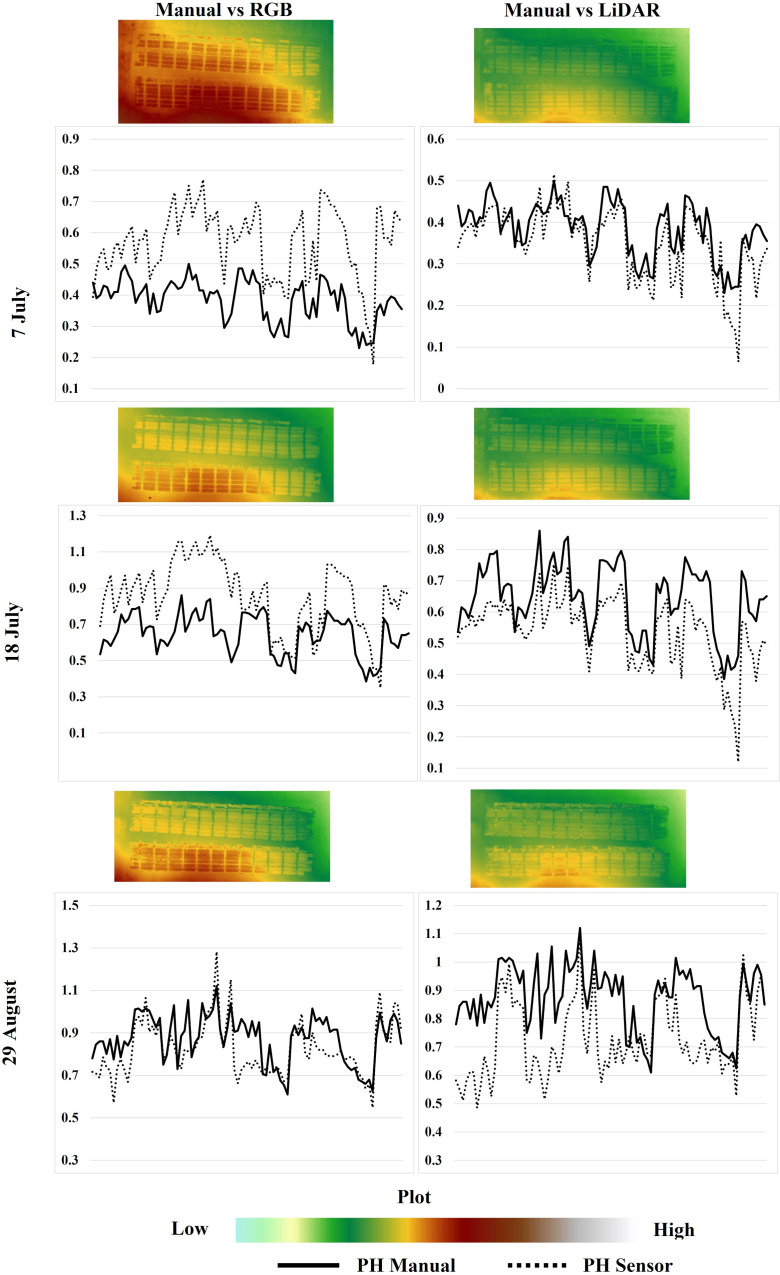
PH distribution among 88 plots across three dates showing ground reference height by the solid line and sensor-based height by dotted line. The 2D plots were generated using ArcGIS Pro for RGB and LiDAR images.

The PH measured manually in the field using a ruler was similar to the values estimated using aerial sensors mounted in the drones. The comparison can be seen in **Table 4**, where the distribution of the height values among different sensors and ground reference height is shown. The pattern of PH distribution, in general, is similar on all three dates. However, a considerable variation in the PH distribution can be noticed on the 7th of July and 18th of July in the case of RGB vs. Manual and the 29th of August in the case of LiDAR vs. Manual PH comparison. Furthermore, the complete distribution of ground reference PH and the PH collected using different aerial sensors across different dates can be visualized in **Figure 6**.

**Table 4:** Average PH using different methods across different aerial dates of soybean.

	7-Jul			18-Jul			29-Aug		
	Manual	RGB	LiDAR	Manual	RGB	LiDAR	Manual	RGB	LiDAR
PH (m)	0.39	0.57	0.35	0.65	0.85	0.54	0.87	0.82	0.73

**Figure 7:**
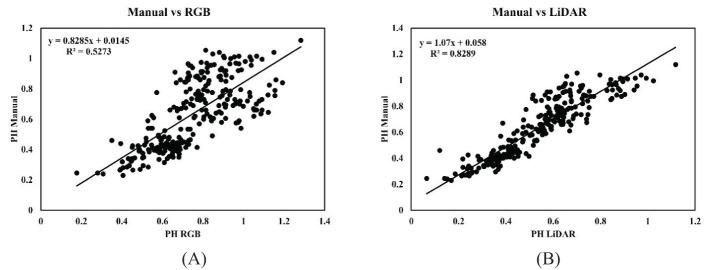
Linear relationship between PH estimated using a ruler in the field and the PH measured using **(A)** RGB camera and the **(B)** LiDAR sensor throughout the season.

A simple linear regression analysis assessed the relationship between PHs derived from Canopy Height Models (CHM) using RGB and LiDAR sensors and the heights measured manually in the field across different dates. The ground reference PH and RGB-derived PH showed a moderate correlation with the coefficient of determination (R^2^) equal to 0.52. The comparison between manually measured soybean height against the LiDAR-derived PH showed a strong correlation with the coefficient of determination (R^2^) of 0.82. Scatterplots illustrating these comparisons are displayed in **Figure 7**: **Figure 7A** for UAV-based RGB vs manually measured PH, and **Figure 7B** for UAV-based LiDAR vs manually measured PH across various stages.

**Figure 8:**
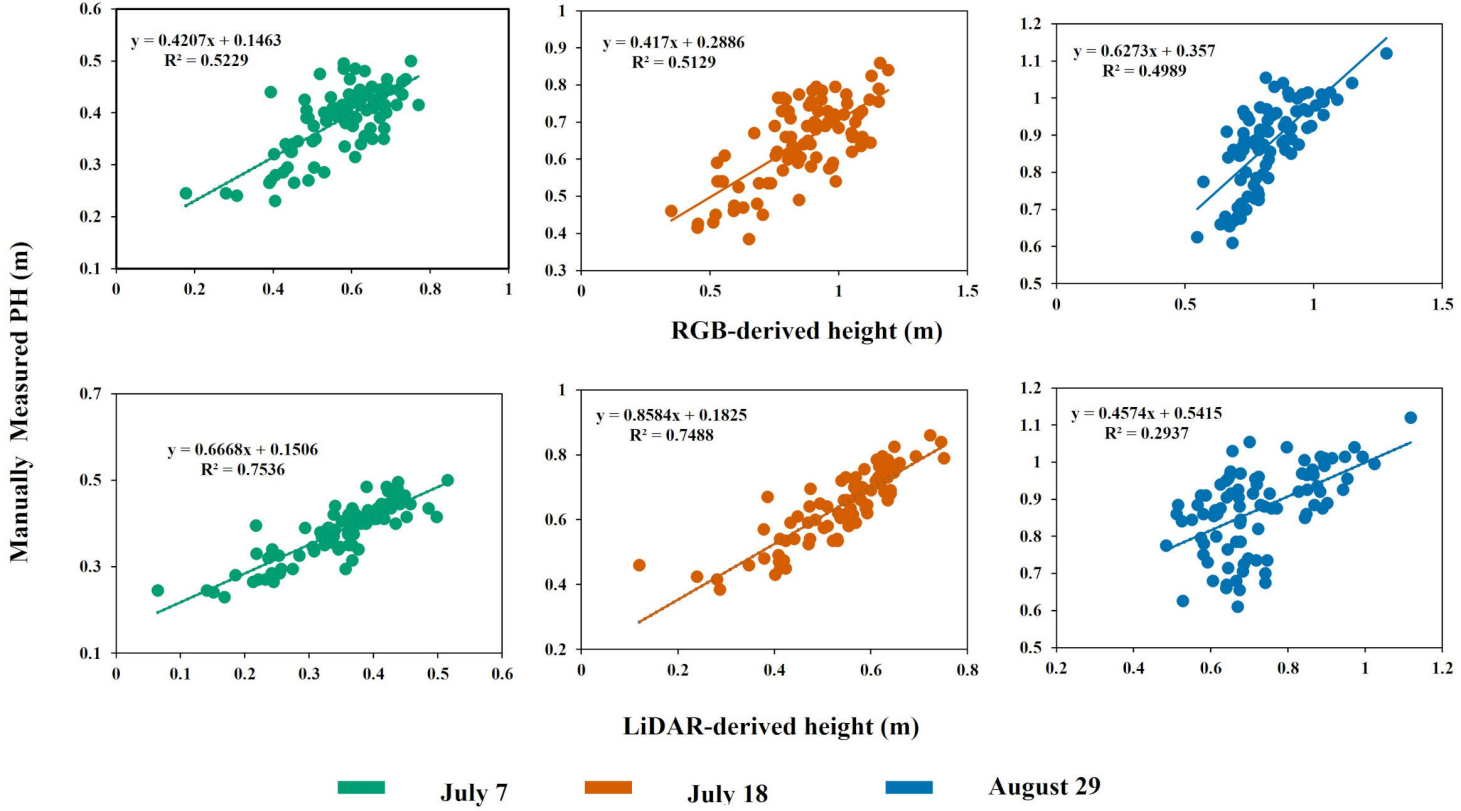
Comparison of the manually measured PH and estimated PH using aerial (top) RGB and (bottom) LiDAR sensor.

In the case of manually measured PH versus RGB-derived PH, the coefficient of determination (R^2^) value ranged between 0.52 and 0.49 in decreasing order across July 7, July 18, and August 29. The correlation remained relatively consistent on July 7 and July 18, with an R^2^ of approximately 0.52. On August 29, the correlation decreased slightly with an R^2^ of 0.49. The PH distribution pattern was similar to RGB vs. manual PH measurement when comparing ground-measured and LiDAR-generated PH. The coefficient of determination (R^2^) between 0.75 and 0.29 was recorded across various dates in descending order, as shown in **Figure 8**. On July 7, the highest R^2^ value was recorded; however, by August 29, the correlation dropped significantly to an R^2^ of 0.29, suggesting that the precision of LiDAR may decrease as the crop progresses toward physiological maturity. Looking specifically at the RMSE and MAE values, as shown in **Table 5**, the comparison between manual measurements and LiDAR-based PH showed the lowest values on July 7. In contrast, the comparison between RGB-measured PH and ground reference PH on July 18 generated the highest RMSE and MAE values, indicating a lesser agreement between ground reference height and RGB-derived height at that particular stage of the crop. The lesser RMSE and MAE values indicate the substantial agreement between the ground reference PH and sensor-measured height. Despite the inharmonious correlation values between the PH generated using aerial sensors and ground reference PH, their relationship remains statistically significant (p < 0.001) across all the growth and developmental periods.

**Table 5:** Comparative result between RGB and LiDAR sensors against manual measurements.

	RGB vs. Manual			LiDAR vs. Manual		
	7 July	18 July	29 August	7 July	18 July	29 August
R^2^	0.52	0.51	0.49	0.75	0.74	0.29
RMSE (m)	0.2	0.24	0.11	0.05	0.12	0.18
MAE (m)	0.18	0.21	0.08	0.04	0.11	0.15
T	9.7	9.5	9.2	16.2	15.9	5.9
P	***	***	***	***	***	***

***, **, * indicate significance at 0.001, 0.01, and 0.1 levels respectively.

**Figure 9:**
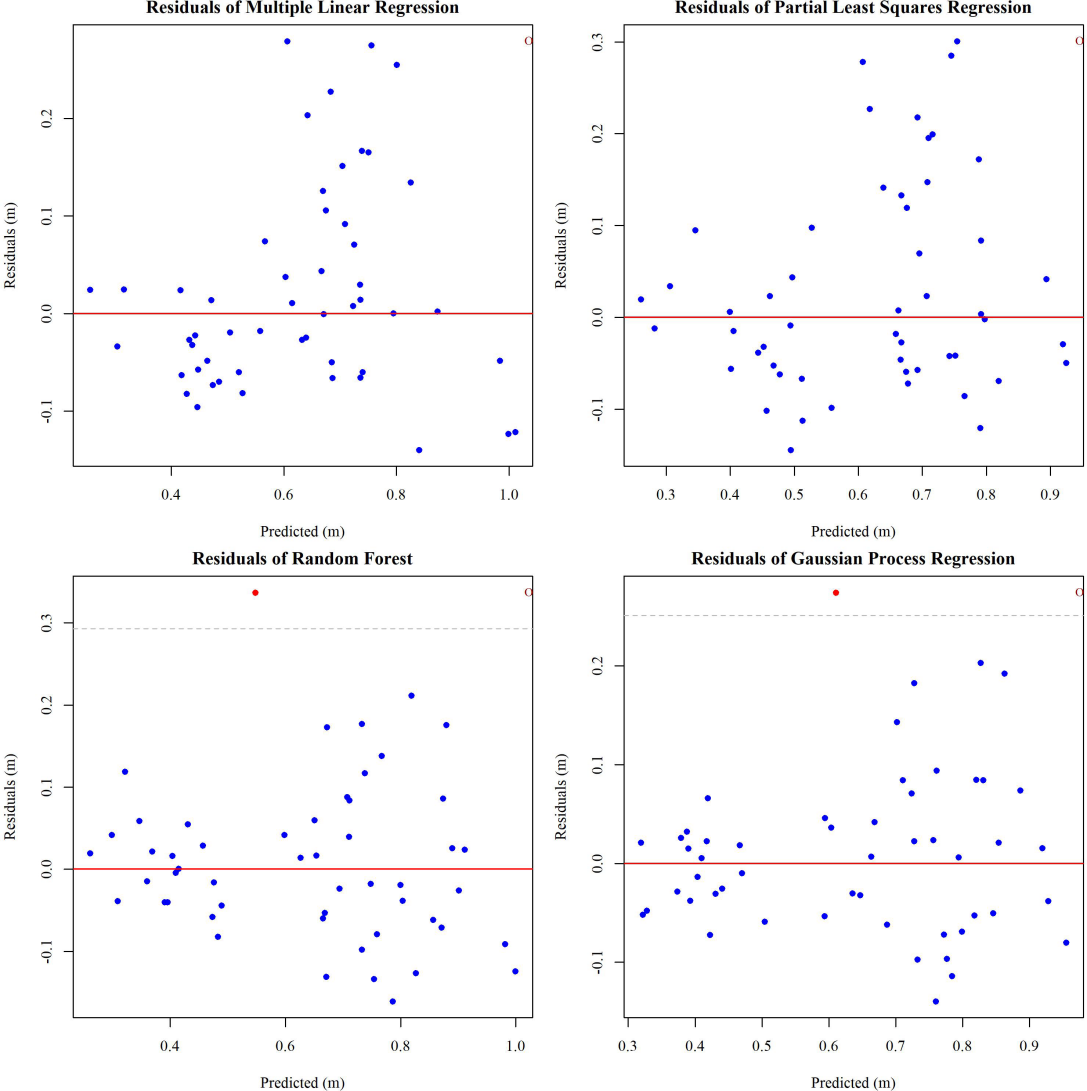
Residual plots for different regression models with ±3 standard deviation thresholds.

Various regression models were employed to evaluate the accuracy and effectiveness of LiDAR and RGB sensors in predicting soybean PH. Meanwhile, outliers were identified using residual diagnostic as shown in **Figure 9**. They were included in the analysis unless they exceeded 3 standard deviations, as their effect on the model was minimal.

**Figure 10:**
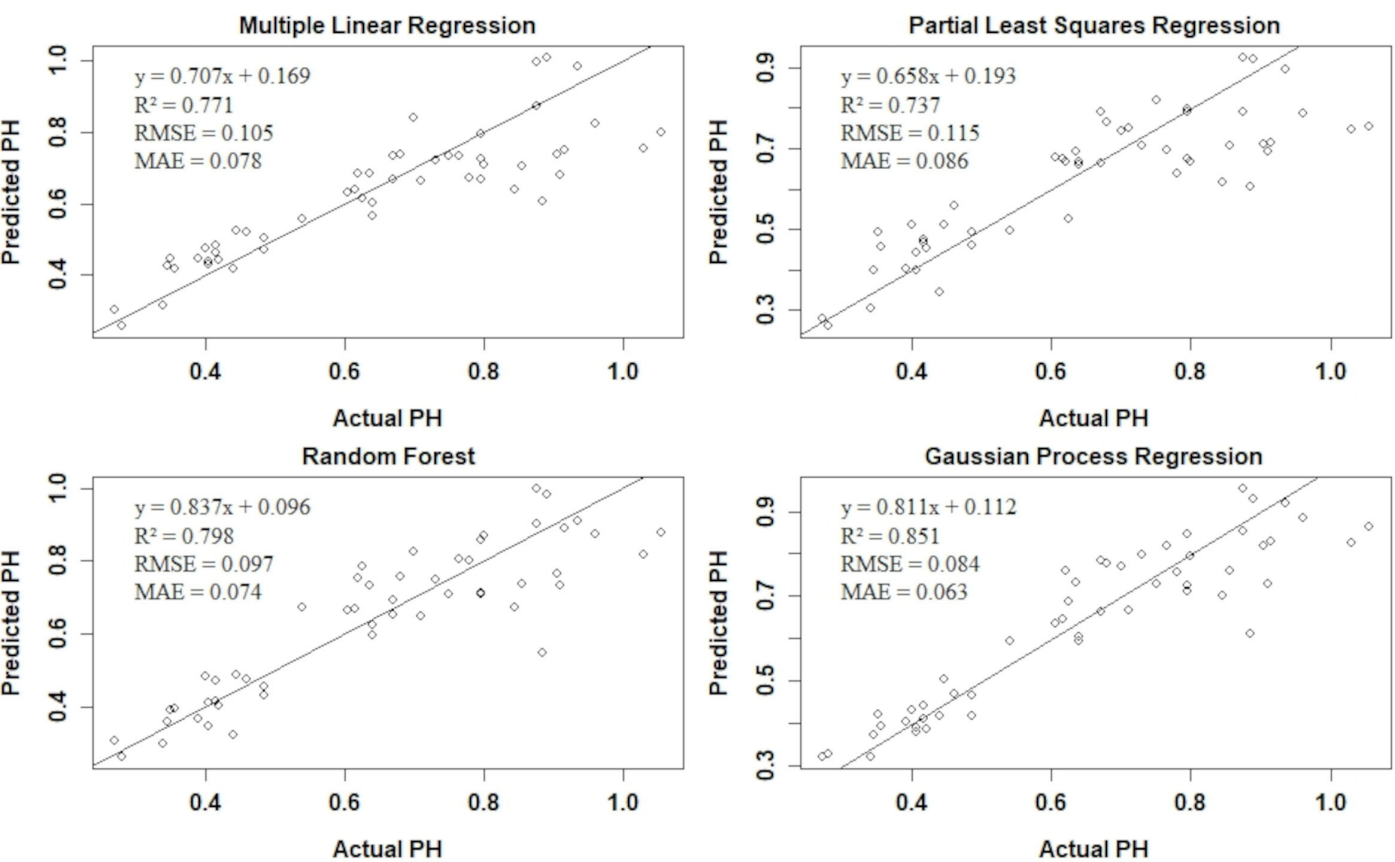
Performance metrics for different regression models (Multiple linear regression, partial least square regression, random forest, and Gaussian process regression) on the test data set.

To evaluate the performance of our regression models in predicting PH, we calculated the R^2^, RMSE, and MAE on the test dataset summarized in the **Figure 10**.

The GPR model exhibited the highest R^2^ value of 0.85, indicating that it explains 85% of the variance in the PH data. Additionally, GPR has the lowest RMSE and MAE, suggesting superior predictive accuracy and consistency compared to the other models. The RF model also performed well, with an R^2^ of 0.79, RMSE of 0.09, and MAE of 0.07. The MLR model achieved an R^2^ of 0.77, RMSE of 0.1, and MAE of 0.078, indicating reasonable performance with higher prediction error compared to GPR and RF. The PLSR model had the lowest performance, with an R^2^ of 0.73, RMSE of 0.11, and MAE of 0.08.

To assess the generalizability of the models, 5-fold cross-validation was performed, with the results summarized in **Table 6**. The cross-validation metrics provide a more robust estimate of the model performance by averaging the result across multiple folds. The RF model maintained strong performance in cross-validation, with a mean R^2^ of 0.85, RMSE of 0.09, and MAE of 0.06. The GPR model showed consistent results with a mean R^2^ of 0.8, RMSE of 0.11, and MAE of 0.08, demonstrating its reliability and stability.

**Table 6:** Summarized result of 5-fold cross-validation metrics.

Model	R^2^ Mean	R^2^ SD	RMSE Mean	RMSE SD	MAE Mean	MAE SD
MLR	0.84	0.01	0.09	0.002	0.07	0.002
PLSR	0.78	0.021	0.1	0.004	0.08	0.004
RF	0.85	0.02	0.09	0.006	0.06	0.005
GPR	0.8	0.03	0.11	0.006	0.08	0.008

The MLR model achieved a mean R^2^ of 0.84, RMSE of 0.09, and MAE of 0.07, indicating good performance with low variability, as reflected by the slight standard deviation. The PLSR model had a mean R^2^ of 0.78, RMSE of 0.1, and MAE of 0.08, showing slightly lower performance compared to the other models but with acceptable stability.

## 4 Discussion

This study focused on identifying the most effective sensor between the two most widely used sensors (RGB camera and LiDAR) for PH estimation using UAVs. Across the crop growth cycle, HTAP was conducted at three different times. Our result showed that PH estimation in soybean using UAV-based LiDAR (R^2^ = 0.83) could be the most reliable than the UAV-equipped RGB camera (R^2^ = 0.53) in the pod growth and seed filling stages. However, the result showed the reliability of deploying RGB cameras, specifically in the physiological maturity stage when LiDAR cannot capture highly correlated results. Similarly, the study highlighted factors like scan angle and elevation adjustment critical in canopy height generation using aerial platforms.

### 4.1 Estimation of crop height

In this study, we assessed crop height using RGB and LiDAR sensors across three soybean growth stages. To enhance the precision of crop height predictions from RGB imageries, we adjusted the DTM and DSM rasters by applying elevation adjustment. These adjustments used offset values calculated from buffers around GCPs and checkpoints. For LiDAR point clouds, we set appropriate scan angles and performed ground classification using the multiscale curvature classification (MCC) method, which effectively distinguishes between ground and non-ground points. The MCC algorithm, along with the Progressive morphological filter (PMF) and cloth simulation function (CSF), was evaluated to refine the DTM accuracy. PMF, as described by Zhang et al. (2003) classifies points as ground and non-ground points based on a dual-threshold approach. Similarly, CSF, as explained by Zhang et al. (2016) simulates a virtual cloth dropped over an inverted point cloud to identify ground points, and MCC uses curvature thresholds to interpolate the ground surface as explained by Evans and Hudak (2007). The PMF algorithm might remove essential terrain details by classifying ground points as non-ground (Zhang et al., 2003). Similarly, the CSF algorithm struggles with the classification of low vegetation and MCC is well-suited for the classification of complex vegetated surfaces (Roberts et al., 2019). In our study, ground classification was done during the extraction of the DTM dataset at the early growth stage of soybeans, so MCC was preferred over CSF to obtain accurate terrain information.

For UAV-based data collection, RGB cameras offer a cost-effective solution despite their limitations in penetrating dense canopies (Cao et al., 2021; Luo et al., 2021). The images can also be processed in user-friendly processing software. The structure from motion (SfM) photogrammetry technique is used to gather canopy height and structure details from high-resolution images as this method can generate a point cloud from several images (Kalacska et al., 2017; Coops et al., 2021). UAV-based RGB camera estimated PH and proved as an important proxy for dry biomass in summer barley (Bendig et al., 2015). UAV-based imaging measurement system quantified PH with minimum error in cotton (Feng et al., 2019). PH and leaf area index (LAI) of different soybean varieties were estimated using a Kinect 2.0 sensor indoors (Ma et al., 2019). Conversely, LiDAR technology excels by penetrating crop canopies to measure PH accurately, unaffected by external lighting (Yuan et al., 2018). The advantageous features of LiDAR include its ability to penetrate the crop canopy, enabling it to reach the ground (Dalla Corte et al., 2022) and supply 3D structural information invaluable for HTAP (Parker et al., 2004; Omasa et al., 2006). Terrestrial laser scanning (TLS) produced promising PH and showed its potential for non-destructive biomass estimation in maize (Tilly et al., 2014b) and rice (Tilly et al., 2014a). Multiple sensors mounted on commercial wild blueberry harvesters proved very efficient at estimating PH and fruit yield (Farooque et al., 2013). A study on winter wheat using a field phenomics platform (FPP) of LiDAR and a time of flight (ToF) camera produced a strongly correlated PH with manual height (Zhou et al., 2015). UAV-LiDAR was effective in estimating PH in sugar beet and wheat, while it was difficult in potatoes due to the complex canopy structure and uneven terrain created by ridges and furrows (ten Harkel et al., 2019).

Our study found that the UAV-mounted LiDAR more accurately predicted soybean height which aligns with similar conclusions in other crops like wheat (Madec et al., 2017; Jimenez-Berni et al., 2018; Yuan et al., 2018), sorghum (Maimaitijiang et al., 2020), and maize (Liu et al., 2024). In our study, there was a strong correlation between the PH obtained from the LiDAR sensor and manual measurement on July 7 and July 18. The R^2^ values obtained in all three growth stages were greater than those of the earlier study done by Luo et al. (2021) using UAV-LiDAR in soybean. This may be attributed to the overestimated DTM, which led to a smaller correlation value in the earlier studies. To avoid DTM overestimation, our study used aerial data when the soybean field was completely visible, and soybean were in a very small growth stage. We observe a decline in R^2^ value in the R7 stage which aligns with similar studies in maize by Zhu et al. (2020) where a significant decrease in plant length, PH, canopy height, and plant width was observed as the plant progressed toward maturity and leaves fell off. At the R7 stage, soybeans undergo senescence, leading to leaf drop and changes in canopy structure. These changes might affect the LiDAR’s ability to capture accurate plant height due to reduced canopy density and increased exposure to underlying structure that might have reduced the plot aggregated mean of all the pixels. Photogrammetry PH shows a moderate correlation in all the R3, R5, and R7 stages as demonstrated by moderate R^2^ values. However, increasing RMSE value indicated increasing deviation of RGB-derived PH from manual measurement. Similar results were found in the earlier studies where the CHM created using the SfM technique exhibited some inaccuracies in height measurement, specifically noticeable in shorter plants (Cunliffe et al., 2016; Wijesingha et al., 2019). Our finding of PH obtained from RGB showing moderate correlation aligns with the conclusion from previous research on corn (Grenzdörffer, 2014; Bareth et al., 2016), which indicated that the photogrammetry technique struggles to reconstruct the uppermost parts of the canopy accurately. The overestimation in the PH estimated from the RGB camera in comparison to the LiDAR sensor may be partly due to the disparity in the spatial resolution of the two sensor systems as well as differences in canopy penetration capacity (Madec et al., 2017).

Overall, the regression models validated the PH predictions, with Gaussian Process Regression (GPR) showing the best performance and MLR and PLSR the least. Incorporation of the model improved the soybean height prediction demonstrated by the increased R^2^ from 0.83 (LiDAR) to 0.85 (GPR). A similar result was obtained in the study of PH using UAV-based oblique photography and LiDAR sensor in maize (Liu et al., 2024). This study underscores the effectiveness of integrating multiple sensing technologies and analytical models to optimize the accuracy of crop height assessments throughout different stages of plant growth.

### 4.2 Influence of scan angle in PH prediction using LiDAR sensor

The complex plant morphology makes it harder for laser penetration which makes it difficult to obtain accurate measurements (Saeys et al., 2009). Earlier studies in winter wheat and winter rye demonstrated that overestimation is low for smaller angles and higher for increasing angles (Ehlert and Heisig, 2013). They further concluded the necessity of evaluating the role of scan angle in overestimating the measurement error in individual crop species (Xu et al., 2023) identified and developed a correction model based on scan angle to improve grassland canopy height estimation and demonstrated considerable improvement in PH from their corrected model (Guo et al., 2019) showed how varying scan angles and positions significantly influence accuracy in wheat height measurement throughout its growth stages. Earlier studies using LiDAR technology in predicting PH have not explored the influence of scan angle. However, we observed the distribution of LiDAR pulses across different scan angles and made efforts to identify appropriate scan angles. Reducing the scan angle towards zero can significantly enhance the accuracy of elevation data, particularly in establishing the digital elevation model (Lohr, 1998). To restrict our scan angle ranges to zero we evaluated the distribution of LiDAR pulses across various scan angles and identified that the -15 to 15-degree range consistently captured our ground feature. Our choice of scan angle range was further validated when ground points obtained using the MCC algorithm were uniformly distributed to zero elevation value. Thus, our study identified the optimum scan angle range in soybean PH estimation. Further, studies need to be conducted to quantify the effect of different scan angle ranges on the PH.

### 4.3 Significance of elevation adjustment in PH estimation

In our study, we observed inconsistencies in field elevation, underscoring the necessity of elevation adjustment to enhance the accuracy of PH predictions. PH estimation is particularly susceptible to biases stemming from errors in Digital Terrain Models (DTM) and Digital Surface Models (DSM), as well as the effects of wind (Han et al., 2018). Accurate elevation information is crucial for precision agriculture, as it allows for a detailed understanding of elevation gradients across the research field, which is essential for estimating precise elevation. Despite advancements in remote sensing technologies such as LiDAR and InSAR, which offer improved vegetation height assessments, adjustments for elevation are still required to refine these measurements (Breidenbach et al., 2008). The variability in ground profiles, influenced by external factors and the operation of agricultural machinery, further complicates accurate ground-level detection (Sun et al., 2022). Malambo et al. (2018) also highlight the significant impact that accurate ground surface detection has on PH estimation accuracy.

The role of elevation is particularly critical in ecological studies, such as predicting plant species distribution in mountainous areas, where the pattern of elevation is a key determinant (Oke and Thompson, 2015). Additionally, the creation of DTMs from aerial data, whether from LiDAR or photogrammetric methods, is susceptible to inherent inaccuracies due to sensor noise, atmospheric conditions, or the angle of data acquisition (Hug et al., 2004). Tilly et al. (2015) noted that elevation adjustments are essential when integrating multiple datasets collected at different times or using various technologies, to ensure a consistent reference point across datasets.

Adjusting for elevation not only enhances the precision of PH measurements but also improves the overall interpretation of remote sensing data, facilitating applications such as crop monitoring, yield prediction, and precision farming (Bendig et al., 2014; Mulla and Belmont, 2018). While many studies have acknowledged biases in PH prediction, few have addressed the influence of elevation adjustment as comprehensively as Tilly et al. (2015) and Bendig et al. (2014). In this study, we employed this approach to derive the improved DTMs and DSMs, leading to improved PH estimations from both aerial RGB and LiDAR sensors. This methodological advancement contributes significantly to the field of precision agriculture by providing more reliable data for crop management and research.

### 4.4 Practical application of the study

RGB cameras and LiDAR are the two most popular sensors used for high-throughput plant height estimation techniques across agricultural operations on proximal or aerial platforms. Multiple studies across various crops have already demonstrated the usefulness of both the sensors for reliable plant height estimation. Those results have been valuable sources for farmers, agronomists, and breeders for high throughput plant phenotyping. In the case of soybeans, there are fewer studies regarding UAV-based plant height estimation techniques. The results of this study suggest LiDAR as the most effective sensor for soybean plant height estimation between pod development to seed filling. However, low-cost RGB cameras was found more effective to predict plant height at a later stage (onset of physiological maturity). Thus, the experimental results from this study would be useful to the agricultural researchers and farmers for the selection of the most effective sensor for plant height estimation during different growth stages in soybeans. Recording plant height at an appropriate time using the most effective sensor will help farmers make an informed decision regarding crop management, as plant height is one of the most important proxies for estimating soybean yield and biomass.

## 5 Conclusion

This study explored low-cost RGB and LiDAR sensors (the most popular for PH studies) to evaluate which sensors produced more effective results for the PH estimation in soybean. An appropriate scan angle range was identified during the data processing, and ground classification was done using the MCC algorithm to compute precise DTM values. The CHM-based PH obtained from RGB and LiDAR sensors was compared with ground reference PH collected manually. Low-cost RGB cameras showed a moderate and consistent correlation across all three growth stages. In contrast, LiDAR demonstrated superior accuracy for soybean height estimation. However, aerial data collection timing and scan angle could significantly influence the result. Furthermore, low-cost RGB cameras could still be a more reliable option than LiDAR sensors for estimating soybean height at a later stage. This study verified the potential of low-cost RGB cameras and LiDAR in assessing soybean PH at different growth stages. The results from this study would help select appropriate aerial phenotyping sensors for estimating PH during different soybean growth stages.

## Data Availability

The original contributions presented in the study are included in the article/supplementary material. Further inquiries can be directed to the corresponding author.
